# Pilot study evaluating tolerability and changes in fecal microbiota associated with novel probiotic administration to dogs with diarrhea

**DOI:** 10.3389/fvets.2025.1720932

**Published:** 2026-01-06

**Authors:** Jessi Doshier, Brooke Anderson, Fan Yang, Samuel D. Stewart, Kayla A. Calapa, Rachel Cooper, Heather Wilson-Robles, Mallory Embree, Chand Khanna

**Affiliations:** 1 Veterinary Specialty Hospital of San Diego, Internal Medicine, NVA/Ethos, San Diego, CA, United States; 2 Native Microbials, San Diego, CA, United States; 3 Ethos Discovery, Woburn, MA, United States; 4 Massachusetts Veterinary Referral Hospital, Internal Medicine, Woburn, MA, United States

**Keywords:** chronic, diarrhea, dog, microbiome, microbiota, probiotic, tolerability

## Abstract

**Background:**

Diarrhea is one of the most common reasons for visiting canine veterinary clinics or emergency centers. Common treatment approaches include dietary modification, antibiotics, and/or probiotics, which are frequently initiated empirically. Antibiotics can have detrimental long-term effects on the gut microbiome and contribute to antimicrobial resistance, prompting a need for alternative therapies. Probiotics are a promising option; however, their strain-specific effects on the canine gut microbiome have been insufficiently characterized *in vivo*, particularly in dogs with diarrhea.

**Hypothesis/objectives:**

This study aimed to evaluate tolerability and changes in fecal microbiota in dogs with diarrhea during the administration of a novel, advanced microbiome-derived probiotic (AMP) consisting of live *Peptacetobacter hiranonis*, *Megamonas funiformis*, and *Enterococcus faecium*, strains of which were all originally isolated from the feces of a healthy dog.

**Animals:**

This single-arm, prospective observational pilot study consisted of 11 client-owned adult dogs of various breeds presenting for chronic diarrhea (>5 days) with a Purina Fecal Score (PFS) between 4 and 7.

**Methods:**

Tolerability of the AMP was assessed through serial clinical examinations and comparison of PFS to baseline. Dogs were classified as responders if their PFS improved to <4 by day 7, and as non-responders otherwise. Fecal samples collected at baseline, day 7, and day 56 of AMP administration underwent Illumina amplicon next-generation sequencing (NGS) of 16S rRNA gene fragments (V4 region) to assess the fecal microbiome composition and diversity in each patient.

**Results:**

No adverse events were noted in any dogs receiving the AMP. Clinical improvement in diarrhea was noted in eight of 11 dogs after administration of the AMP. Increases in fecal microbiome alpha-diversity were observed after 1 week of AMP administration for six out of seven long-term participants.

**Conclusion and clinical importance:**

This pilot study indicates that the AMP was well tolerated in dogs with diarrhea, with dogs maintaining or improving clinical appearance during administration. These preliminary findings justify larger controlled studies to evaluate AMP efficacy and to explore associations between treatment, fecal microbiome changes, and clinical response.

**Clinical trial registration:**

Identifier: VCT23005615.

## Introduction

Diarrhea is one of the most common clinical signs exhibited by dogs presenting to a veterinary clinic. Treatments are frequently initiated empirically and may include dietary modifications, antimicrobials, prebiotics, and probiotics. In most cases of diarrhea, the gut microbiome is negatively affected, leading to a perpetuating cycle of inflammation and persistent diarrhea (1). Some commonly prescribed treatments, such as antimicrobials, have been shown to have long-term impacts on the gut microbiome and contribute to antimicrobial resistance (1, 2). This supports the need for alternative therapies and more judicious antimicrobial use. Novel approaches that target the microbiome with greater precision are needed to therapeutically disrupt this cycle, improve patient outcomes, and decrease potential long-term adverse effects of other commonly utilized therapies.

The gut microbiome is a complex population of microorganisms that inhabit the gastrointestinal tract and contribute to gastrointestinal health. The bacterial component comprises the largest portion of this population, playing key roles in maintaining gastrointestinal function and immune regulation. Commensal bacteria influence local and systemic immune responses (3) and help prevent colonization by pathogenic microorganisms (1, 4). They also produce metabolites that can influence gut physiology, including short-chain fatty acids involved in the regulation of satiety, gut motility, and gut epithelial tight junction maintenance (5), and they can lower intestinal pH to inhibit pH-sensitive pathogens (5). In addition to immune and barrier functions, the gut microbiome also contributes to host nutrition. Gut microbial taxa synthesize necessary nutrients that dogs cannot produce, including essential amino acids, B vitamins, and vitamin K (6). These microbial-derived nutrients and metabolites have been linked to overall gastrointestinal health, and their absence or reduction is associated with impaired gut function (7).

Dogs with both acute and chronic diarrhea (>5 days duration) have been found to have an altered gut microbiome, and acute uncomplicated diarrhea (AD) has been associated with microbiome dysbiosis (1). Dysbiosis is defined as a distinct change in the composition and structure of the gut bacterial community (5). It is speculated that inflammation increases intestinal permeability and local oxygen availability and leads to overgrowth of facultative anaerobes and dysbiosis (1). Gut microbial populations, especially obligate anaerobes that are killed in the presence of oxygen, are negatively affected as a result (1). Overpopulated species such as C. difficile can produce toxins that impair tight junctions, along with other negative bacterial metabolites that cause osmotic/secretory diarrhea (3, 8). Underpopulated species such as *P. hiranonis* lead to reduced production of secondary bile acids, which have activity against pathogenic species (3). Diarrhea is also associated with a decreased abundance of short-chain fatty acid-producing bacteria and with metabolites associated with immune pathways, changes that may lead to increased inflammation (3). With these changes, there is a disruption in the intestinal mucus layer, which can also lead to pathogen adherence or invasion and an imbalance of bacterial species (3). Acute and chronic diarrhea are often accompanied by reduced microbial diversity, shifts in the relative abundances of commensal taxa, and overgrowth of pathogenic microorganisms.

Probiotics, a common therapy for dogs with diarrhea, are live microorganisms administered orally with the intention of modifying the gut ecosystem and microbial composition. Reported benefits are strain-specific, and they are easy to administer, safe, and relatively inexpensive for most dog owners, with only rare adverse effects. In contrast, antimicrobials have been suggested to induce dysbiosis in both human and experimental animal studies (2). Antimicrobial-induced alterations of gut microbial metabolites, such as bile acids and short-chain fatty acids, can further exacerbate intestinal inflammation (2). Probiotics can influence immune activity, produce antimicrobial compounds, and promote normalization of host microbiota (9, 10). They have been shown to improve barrier function and help modulate the number of mucosal bacteria, maintaining mucosal homeostasis (11). However, data supporting probiotic use in veterinary medicine remains limited, and it is difficult to determine which patients may benefit from which specific strains. Although probiotics are commonly recommended, several studies in humans suggest that certain commercial formulations can slow the natural recovery of the host microbiome after antimicrobial exposure (12–14). This delay may occur because administered strains compete with native taxa that are attempting to reestablish their normal structure. These findings underscore the importance of evaluating strain-level activity in dogs rather than assuming that all probiotic products support microbiome restoration. Probiotics remain an active area of investigation, and further work is needed to clarify their role for dogs with diarrhea (15).

*In vivo* probiotic use in dogs remains understudied, especially the use of strains naturally found in the canine gut and their impacts on the gut microbiome. The majority of commercially available probiotics for dogs contain a limited range of species, including *Lactobacillus* and *Enterococcus* spp., which are typically derived from non-canine sources. Such strains may have reduced ability to establish colonization or exert meaningful functional effects compared with host-adapted microbes (16). Sequencing-based microbiome surveys of healthy dogs have identified candidate taxa that are likely important to gut health, but few have been developed into live biotherapeutics. The advanced microbiome-derived probiotic (AMP) in this study contains three canine microbiome-derived strains: Clostridium hiranonis ASCUSCA136, which has since been reclassified as *Peptacetobacter hiranonis* ASCUSCA136 (17), *Megamonas funiformis* ASCUSCA104, and *Enterococcus faecium* ASCUSCA103. The AMP strains were selected from an analysis of large microbiome sequencing datasets according to their potential to restore microbiome balance in dogs with diarrhea and gastrointestinal dysbiosis (18). In brief, to qualify for inclusion in the probiotic, a taxon had to be common among the gut microbiome sequences of healthy dogs, positively correlated with other core gut microbiome species, and negatively correlated with diagnoses of chronic enteropathies. All three selected strains have been independently identified in correlation with health states in dogs and have been studied for their roles in secondary bile acid production (3), fiber digestion (19), and gut immune system activation (9).

These three live microbial strains, originally isolated from the feces of a healthy dog, have never been employed for use as a probiotic in dogs. This study is intended to provide an evaluation of the tolerability of this novel three-strain AMP in dogs experiencing diarrhea and is a single-arm, uncontrolled pilot study that was not designed to evaluate efficacy. The primary endpoint of this study was to assess tolerability via observation of any adverse effects. Secondary endpoints for this study were to describe the changes in the fecal microbiome composition and diarrhea severity following administration of the AMP. Because the microorganisms in this probiotic are native to the canine gut, we hypothesized that the AMP would be well-tolerated and that changes in fecal microbiome composition would fit with previously observed trends (1).

## Materials and methods

### Study design and approval

This study was designed as a prospective, single-arm, open-label, multicenter pilot study to assess the tolerability of a novel advanced microbiome-derived probiotic (AMP) in client-owned dogs with diarrhea. The study was conducted following ethical approval from the ACI Biosciences Animal Care and Use Committee (Chevy Chase, MD). Eligible dogs were enrolled with the owner’s informed consent.

The data collected from this study will be used to support future studies to further investigate the clinical efficacy of the AMP.

### Animals and enrollment criteria

Dogs were enrolled through an urgent care clinic (Boston, MA) and a general practice (Tulsa, OK) between August 2022 and February 2023. Eligibility criteria included age greater than 1 year and Purina Fecal Score (PFS) of ≥4, with diarrhea classified as large bowel, small bowel, or mixed. Dogs whose diarrhea did not resolve on a bland diet were eligible. All breeds and sexes of dogs were eligible.

Exclusion criteria were hemorrhagic diarrhea of more than seven days duration, systemic antimicrobial treatment within 1 week before study enrollment, PFS between 1 and 3, or a positive fecal float or Giardia ELISA.

Dogs were removed from the study if they received any of the following concomitant treatments during the study period: antimicrobials, antifungals, other probiotics, corticosteroids, chemotherapeutics, non-steroidal anti-inflammatories (NSAIDs), or antacids. Changes in diet or the occurrence of a serious adverse event also led to removal.

### Baseline evaluation and sample collection

At the baseline visit (day 0), each dog underwent a physical examination, documentation of current medications and diet, and assignment of a PFS. Two grape-sized fecal samples were collected manually with a fecal loop or after natural elimination. One sample was placed in a standard collection container for fecal float and Giardia ELISA and stored in a refrigerator (4 °C). The second sample was placed in a 50-mL conical tube for baseline microbiome analysis via next-generation sequencing (NGS) and stored in a freezer. The samples were initially stored in a − 20° C freezer in each hospital immediately following collection. Once they were received after being shipped out, they were then stored at −80° C for the remainder of the time until they were analyzed. If stool was unavailable, a fecal swab was collected and stored in a white top tube without additives for microbiome analysis. Blood was also collected in clot activator tubes, frozen, and submitted to Texas A&M University’s Gastrointestinal Laboratory for serum assays: canine trypsin-like immunoreactivity (cTLI), cobalamin, folate, and canine pancreatic lipase immunoreactivity (cPLI). Serum assay results were evaluated using the laboratory’s reference intervals.

### AMP administration

Eligible dogs began a treatment regimen with a fixed dose of ¼ tsp (0.70 g) for patients weighing less than 40 pounds and ½ tsp. for patients weighing 40 pounds or more. The dogs received the treatment once daily with food. The AMP contained three canine-derived bacterial strains: 1E7 CFU/g *Peptacetobacter hiranonis* ASCUSCA136, 1E5 CFU/g *Megamonas funiformis* ASCUSCA104, and 1E7 CFU/g *Enterococcus faecium* ASCUSCA103. Owners were instructed to record daily PFS values and report any potential adverse events that could be related to AMP administration.

No standardized body weight-based formula exists for probiotic dosing in dogs. The International Scientific Association for Probiotics and Prebiotics (ISAPP) recommends that dosing should be based on clinically tested doses of specific strains shown to provide the desired benefits in the relevant population (20). A higher dose is not necessarily more beneficial. In this study, the daily dose for each strain was determined using healthy canine microbiome survey data to target reference abundances during administration, in combination with the estimated microbial load in the small intestines of laboratory Beagles. Strain-specific dosages in the range of 0.1–10% of the total microbial cell counts were targeted, with the lower end of the total count estimated at 1.6E8 CFUs based on previously reported microbial densities in the healthy dog small intestine (21) and the small intestinal volume of research Beagles (22), which typically weigh 20–30 lbs. as adults. To account for the size bias inherent to these calculations, the dose was doubled for dogs conservatively considered twice the size of Beagles, at 40 pounds or more.

### Follow-up and response classification

On day 7, the dogs returned for a physical examination, repeated PFS assessment, blood collection for a GI panel, and fecal sampling following the same procedures as at baseline. The dogs were classified as responders if they had a PFS score of 3 or lower and were instructed to continue the AMP until day 56. The dogs with a PFS score of 4 or higher were classified as non-responders and were offered rescue treatment with metronidazole (10–15 mg/kg PO BID). Borderline cases among non-responders, where a dog’s PFS had dropped substantially from baseline but did not meet the PFS threshold to be classified as a responder, were given the option to turn down the rescue treatment and instead continue the study with veterinarian and owner agreement.

PFS assessments were repeated on days 14 and 28 using stool photographs provided by owners. On day 56, all dogs that remained enrolled underwent a final physical examination, PFS assessment, blood collection for a GI panel, and fecal sampling for microbiome analysis. Tolerability was assessed based on voluntary consumption of the AMP and the absence of grade 3 adverse events, as defined by the Veterinary Cooperative Oncology Group—Common Terminology Criteria for Adverse Events (VCOG-CTCAE v2) (23).

### Microbiome sequencing and data processing

Fecal samples for microbial community sequencing were frozen until processing. Genomic DNA was extracted using the Qiagen DNeasy PowerSoil Pro Kit (Qiagen, Hilden, Germany). The 16S rRNA gene (V4 region) was amplified via PCR with Platinum Hot Start Master Mix (Invitrogen, Carlsbad, CA, United States) and barcoded 806R/515F primers with Illumina adapters (Eton Bioscience, San Diego, CA, United States) (24, 25). Fragment size and amplification were confirmed by 2% agarose gel electrophoresis. Amplicons were pooled, purified with AMPure XP beads (Beckman Coulter, Brea, CA, United States), and quantified using the Qubit dsDNA HS assay (Life Technologies, Eugene, OR, United States). Libraries were sequenced on an Illumina MiSeq platform (Illumina, San Diego, CA, United States) using standard protocols outlined for the single-end 300 bp v2 sequencing kit, and raw reads were de-multiplexed using Illumina factory software.

Adapter sequences were removed from demultiplexed sequence reads with Cutadapt (26). All demultiplexed read files were filtered based on quality scores with QIIME 2 (27) version 2023.9 and then passed through the QIIME 2 16S rRNA Deblur workflow for trimming to 250 nucleotides, denoising, and singleton removal. Unique amplicon sequence variants (ASVs) were assigned taxonomy using the QIIME 2 sklearn-based classifier trained with the Silva 138 99% identity non-redundant SSU reference database. ASV raw count tables were grouped by taxonomic ranking and transformed for compositional data analyses using robust centered log-ratios (rCLR) (28).

### Statistical and microbiome analyses

Diversity metrics were calculated using scikit-bio v0.7.0 for the Shannon index and the R package vegan v2.7–1 (29) for beta-diversity metrics and dimensionality reduction. Alpha diversity was assessed over time using linear mixed-effects models to account for repeated measurements from dogs. Models were implemented using the package lme4 v1.1–37 in R (30) with time point encoded as a fixed effect with dog as a random intercept. Pairwise contrasts between time points were evaluated using the emmeans v1.11.2 (31) package with degrees of freedom estimated using the Satterthwaite method to account for small sample sizes. For beta-diversity analyses, sequenced fecal samples from the present study were combined with a publicly available 16S rRNA sequencing dataset (‘reference cohort’; also the V4 region) containing 150 samples from dogs diagnosed with idiopathic irritable bowel disease (IBD) and healthy controls (32). This combined sequencing dataset was trimmed to 90 nucleotides and denoised using Deblur to make cross-cohort comparisons. Beta-diversity was assessed by principal component analysis using a Euclidean distance matrix of rCLR-transformed ASV counts (33). Beta-diversity differences were assessed through PERMANOVA using vegan:adonis.

ASVs matching the microbes in the AMP were identified using VSEARCH v2.27.1 (34) to query fecal ASVs for the V4 region of 16S rRNA sequence for *Megamonas funiformis* ASCUSCA104, *Peptacetobacter hiranonis* ASCUSCA136, and *Enterococcus faecium* ASCUSCA103. Hits with 100% identity across the query sequence length were used for rCLR transformation of the AMP taxon counts within a sample.

Taxonomic strains included in the commonly used Texas A&M Dysbiosis Index (35) were identified by querying sequences in fecal samples using VSEARCH against reference 16S rRNA gene sequences for genera *Fusobacterium, Faecalibacterium, Turicibacter, Blautia,* and *Streptococcus*, and for species *Escherichia coli* and *Peptacetobacter hiranonis*. Minimum identity thresholds were 100% for species-level hits and 99% for genus-level hits to the V4 region of the 16S ribosomal RNA gene, based on the accuracy of taxonomic assignment for this region as evaluated by Edgar (36). Log ratios were calculated as the log of the ratio of the sum of counts for *Fusobacterium, Faecalibacterium, Turicibacter, Blautia,* and *Peptacetobacter hiranonis* to the sum of counts for *Streptococcus* and *Escherichia coli*.

## Results

### Tolerability and adverse events

No adverse events were noted in any dogs receiving the AMP. It was well tolerated in all dogs, and no dose adjustments or patient withdrawals were required due to adverse events.

### Study population

Eleven adult, client-owned dogs with diarrhea were screened and enrolled in the study (Table 1). Eight dogs were neutered males, one dog was an intact male, and two were spayed females. Breeds represented included two American Pitbulls and one each of Australian Shepherd, Basset Hound Mix, Beagle mix, Bernese Mountain Dog, Dachshund, Fox Terrier, German Shepherd Dog, Labrador Retriever, and a Standard Poodle. The median age was 9.5 years (range 2–16 years). The median weight was 57.6 pounds (range 7.6–98.2 pounds). Weight was not recorded for one dog.

**Table 1:** Description of dogs enrolled in the trial.

ID	Breed	Age (years)	Sex	Weight (lbs)	AMP dose (tsp)	Diet
Dog 1	Bassett hound mix	13	MN	28.4	¼	Pure balance salmon and rice
Dog 2	American pitbull	10	MN	71.6	½	Hill’s sensitive skin and stomach
Dog 3	Australian shepherd	7	M	54.8	½	Purina one
Dog 4	Bernese mountain dog	7	MN	92	½	Hills i/d
Dog 5	Labrador retriever	11	MN	63	½	Purina one
Dog 6	Beagle mix	7	FS	-	-	Hill’s sensitive skin and stomach
Dog 7	Standard poodle	2	MN	71	½	Natural balance—Limited Ingredient—Lamb and Rice
Dog 8	German shepherd	7	MN	98.2	½	We Believe Diamond Naturals
Dog 9	Fox terrier	16	MN	7.6	¼	Royal Canin and Hills i/d
Dog 10	Dachshund	13	MN	15.8	¼	Purina Pro Plan Healthy Weight
Dog 11	American pitbull	10	FS	73.4	½	Purina RX Diet OM—Chicken and Rice

AMP dose was stratified by dog weight.

All dogs met the inclusion criteria and tested negative for ova, parasites, and Giardia. Two dogs had benign yeast (Dog 4 and Dog 10; *Saccharomycopsis guttulatus*) observed on fecal float.

### Baseline diarrhea and medications

All dogs had diarrhea classified as “chronic” by the study definition (>5 days duration), though most (*n* = 9) presented with diarrhea for “weeks to months” in duration at enrollment. At the time of the screening, one dog was on Keppra and Cerenia (Dog 3). Another was receiving Cyclosporine ophthalmic drops, Dasequin, and Adequan (Dog 4). The remaining nine dogs were not on any medications or supplements.

### Clinical response

The median PFS at the time of the screening visit was 6.3 (range 4–7). On day 7, seven dogs were classified as responders (PFS ≤ 3) and four were classified as non-responders (PFS ≥ 4). One non-responder continued in the study due to substantial improvement from the start (PFS 7 to 4; dog 10). Among the eight dogs that continued beyond day 7, the median PFS remained under four for the duration of observation (Table 2).

**Table 2:** Purina fecal scores of enrolled dogs on the day of screening, separated by response group, starting day 7.

Day of study	Median purina fecal score (range)		Number patients	
Screening	7 (4–7)		11	
	Non-responder	Responder	NON-responder	responder
7	5 (4–6.5)	3 (2–3.5)	4	7
14	2*	2 (2–4)	1	7
28	3*	2 (2–3)	1	7
56	3*	2 (2–3)	1	7

* No ranges are reported because *n* = 1 for non-responders beyond day 7.

Non-responders who left the study did not receive the planned metronidazole rescue protocol. Instead, one received tylosin, one was instructed to add pumpkin puree to the diet, and one dog was lost to follow-up with no additional information.

### Gastrointestinal panel results

GI panel serum assays were performed at the screening visit (day 0) for all dogs and at days 7 and 56 for most dogs that remained in the study. Dogs 4, 8, and 11 did not have a repeat GI panel performed throughout the study. Dog 6 had one performed on day 7, but not on day 56. Measured serum markers included cTLI and cPLI for evaluation of common pancreas issues and cobalamin and folate for assessment of distal small intestinal malabsorption and/or dysbiosis (Supplementary Figure 1).

No dogs had cTLI values within the diagnostic or equivocal ranges for exocrine pancreatic insufficiency (<7.6 ug/L). Nine dogs were within the reference interval for cTLI (10.9–50.0 μg/L) at screening, and two dogs (Dog 2 and Dog 6) were elevated (>50 μg/L). Dog 6 returned to the normal reference range, but Dog 2 remained elevated through day 56. During treatment, Dog 9 developed elevated cTLI on day 7 and day 56, and Dog 5 developed elevated cTLI on day 56.

Serum cPLI at screening was <200 μg/L for all dogs except for Dog 4 (205 μg/L), who was removed from the study by day 7. Dogs 2 and 9 had elevated serum cPLI on day 7 but returned to the normal reference range by day 56. Dog 1 was within the cPLI reference interval until day 56, when cPLI was tested above the limit of detection (>2000 ng/L).

Baseline serum cobalamin was below the reference interval (251–908 ng/L) or the level where cobalamin supplementation is recommended (<400 ng/L) (37) in five dogs (Dogs 9 and 10: <251 ng/L; Dogs 3,4, and 11: 251–400 ng/L). Dogs 4 and 11 were classified as non-responders and removed from the study by day 7. Dog 9 showed increased serum cobalamin concentration on day 7 (>1,000 ng/L) but dropped back below the reference range again on day 56. Dogs 3 and 10 tested within reference but near the low end on days 7 and 56 (329–436 ng/L). Dogs 3 and 10 also had serum folate concentrations outside the reference range (7.7–24.4 μg/L). Dog 3 was below the reference range at screening (6.6 μg/L) and remained near or below the lower limit during the treatment period. Dog 10 was normal at baseline, tested below range on day 7, then above range on day 56 (51.1 μg/L). No other dogs were observed to have serum folate concentrations outside the laboratory reference range.

### Microbiome results

Fecal samples for microbiome analysis were obtained for eight of the dogs enrolled in the study. Four dogs provided samples at all three time points (Dogs 1, 2, 9, and 10), three dogs provided a baseline sample during screening and one sample during AMP administration (Dogs 3, 5, and 7), and one dog classified as a non-responder (Dog 8) discontinued after baseline sampling.

**Figure 1:**
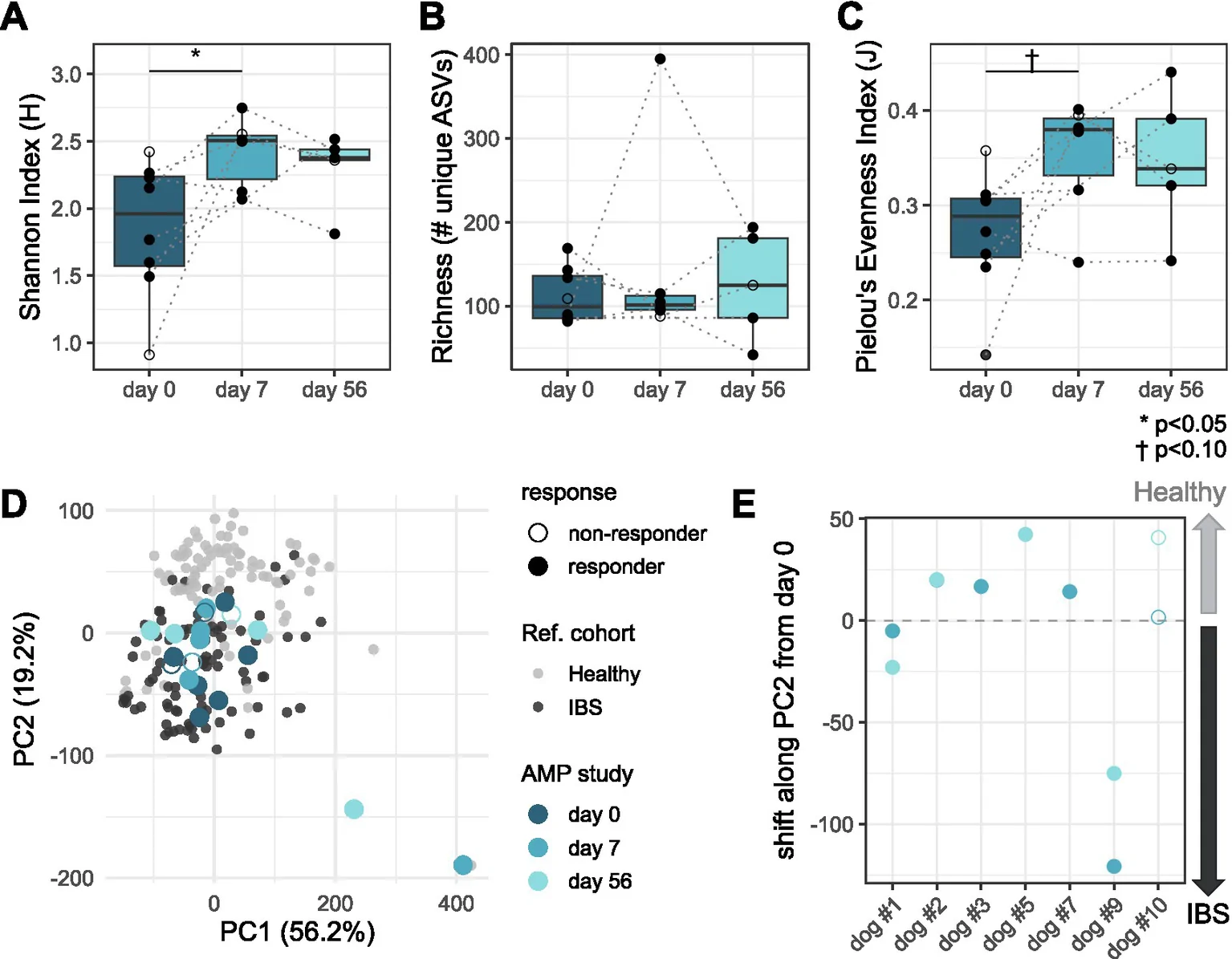
Diversity metrics before (day 0) and during (days 7, 56) AMP treatment. **(A–C)** Estimates of ASV alpha diversity for fecal samples at each time point, with boxplots showing median and first and third quartiles for **(A)** overall diversity changes over time using the Shannon–Weaver index, **(B)** richness of unique ASVs was similar over the study, and **(C)** evenness as measured using the Pielou Index. Dotted lines connect samples collected from the same animal. Differences in diversity metrics between time points were evaluated using mixed effects models accounting for repeated measurements by dog; contrasts tested pairwise differences between time points. **(D)** Principal component analysis of Aitchison distances from fecal samples of dogs enrolled in the present AMP study overlaid on a publicly available reference cohort (“ref. cohort”) of healthy and IBS dog fecal samples (see Methods section). Axis labels report the percent of variance explained by each component. **(E)** Per-dog change along PC2 from the screening visit (day 0) to day 7 or day 56 of AMP treatment; positive shifts indicate movement toward the healthy region of the ordination, whereas negative shifts indicate movement toward the IBS region.

The alpha diversity of fecal samples was found to increase after the initial screening visit, with the median Shannon index moving from 2.0 at screening to 2.5 at day 7 of AMP administration (*p* = 0.046, Figure 1A). Values remained numerically higher on day 56 but were not statistically significant from baseline or day 7. Six of seven dogs who were enrolled for at least 7 days showed an increase in the Shannon index, which was driven by community evenness (*p* = 0.079, Figure 1C) but not richness (Figure 1B). Diversity metrics did not distinguish non-responders from responders.

PERMANOVA provided sample size-limited evidence that samples clustered by dog (*p* = 0.011, betadisper *p* = 0.002). In contrast, samples did not cluster by response classification (*p* = 0.559; betadisper *p* = 0.139) or time point (*p* = 0.840; betadisper *p* = 0.754). To evaluate the changes occurring for each dog individually, fecal samples from this study were merged with a published 16S rRNA dataset of fecal microbiomes from healthy dogs and dogs with IBD (32) and compared using robust Aitchison PCA (Figure 1D). Most of the separation between healthy and IBD dogs was observed along PC2, and five out of the seven study dogs shifted along PC2 toward the healthy dog cluster by day 7 (Figure 1E).

**Figure 2:**
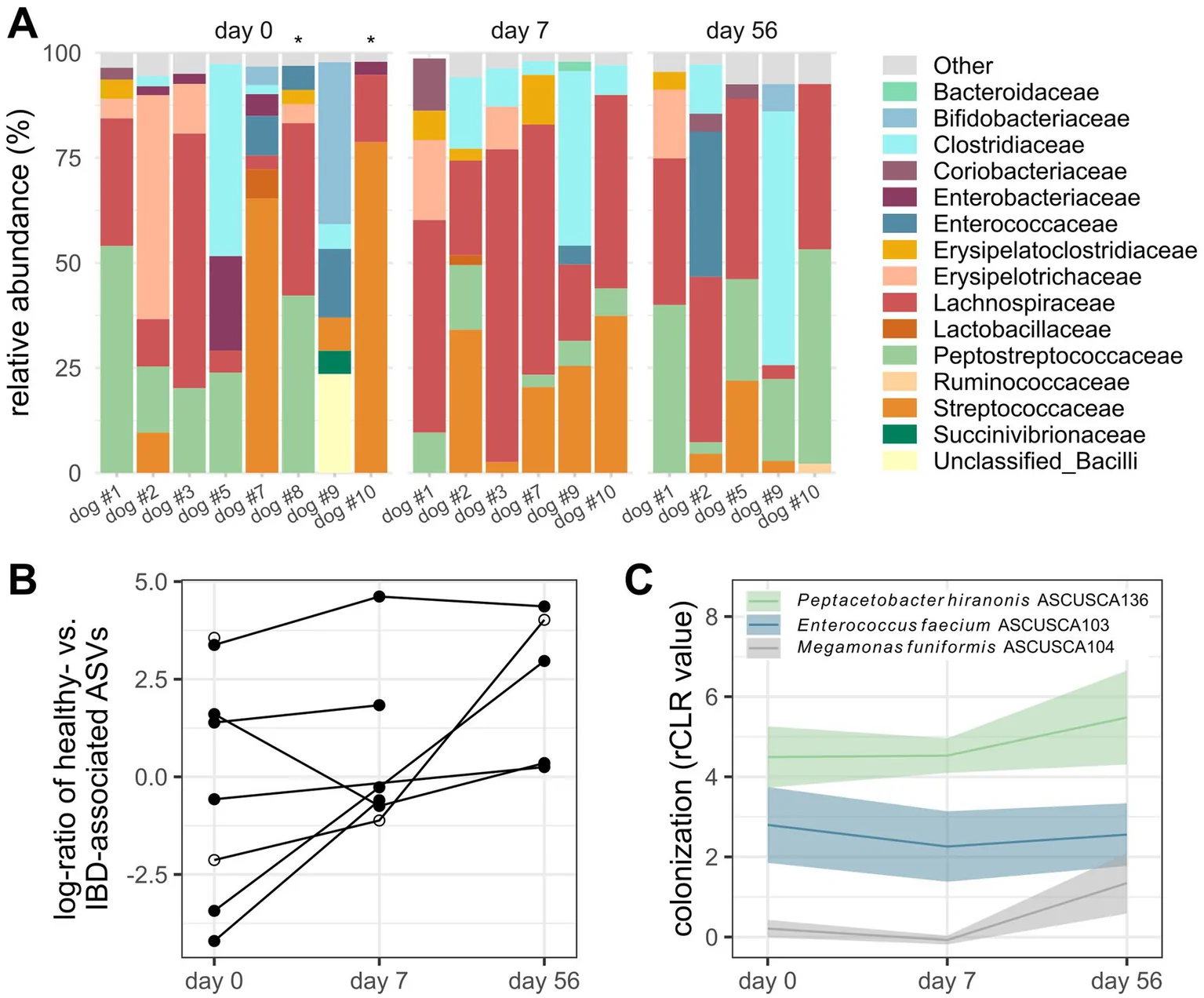
Taxonomic characterization of each dog’s fecal samples at each collected time point. **(A)** Family-level relative abundance of ASVs in fecal samples collected during the screening visit (day 0) and after 7 and 56 days of AMP treatment. Asterisks mark screening samples from dogs later classified as non-responders. **(B)** Changes in the ratio of ASVs that align to taxa used in the dysbiosis index (18), expressed as a log-ratio of ASVs more common in healthy dogs to those more common in CE/IBD dogs (see methods section). The ratio is computed as summed reads from *Fusobacterium*, *Faecalibacterium*, *Turicibacter*, *Blautia*, and *Peptacetobacter hiranonis* divided by summed reads from *Streptococcus* and Escherichia coli. Open symbols indicate non-responders. **(C)** Abundance of the administered AMP strains, quantified as the mean rCLR-transformed value of ASVs with 100% identity to the sequenced 16S rRNA gene region of each canine-derived strain included in the AMP.

The taxonomic makeup of dog fecal samples was diverse and observed to change over time (Figure 2A). Fecal samples collected at screening were particularly variable, with the most abundant microbial family being either *Lachnospiraceae*, *Peptostreptococcaceae*, *Erysipelotrichaceae*, *Clostridiaceae*, *Bifidobacteraceae*, or *Streptococcaceae*. By day 7, the relative abundance of *Lachnospiraceaea* and *Clostridiaceae* members had increased in most dogs, and the relative abundance of *Enterbacteriaceae* and *Bifidobacteraceae* had decreased. By day 56, the relative abundance of *Peptostreptococcaceae* increased in most dogs, while *Strepococcaceae* decreased across most dogs when compared to day 7.

Many of these abundant taxa are measured in the Texas A&M Dysbiosis Index (35). Using the fecal microbiome sequencing data, a ratio of health-associated taxa to chronic inflammatory enteropathy (CE)-associated was calculated based on the taxa utilized in the Dysbiosis Index. Dogs starting with a negative ratio (indicating a greater proportion of taxa associated with CE) were observed to shift toward positive ratios (indicating a greater proportion of taxa associated with a healthy state), while those starting with a positive ratio tended to remain the same (Figure 2B).

Gene fragments of the 16S rRNA sequences for the AMP strains fed in the study were identified in fecal samples taken at screening and in samples taken during the treatment period (Figure 2C). ASVs matching *Megamonas funiformis* ASCUSCA104 and *Peptacetobacter hiranonis* ASCUSCA136 were both found in higher levels at day 56, while *Enterococcus faecium* ASCUSCA103 remained at similar abundance from screening and through treatment.

## Discussion

In this pilot study, the AMP was well-tolerated during a 60-day treatment period with no adverse events reported. This formulation contains three bacterial species isolated from the feces of healthy dogs (*Peptacetobacter hiranonis* ASCUSCA136*, Megamonas funiformis* ASCUSCA104, and *Enterococcus faecium* ASCUSCA103) along with inulin and nutritional yeast flakes for bulk and palatability. These microbes were selected from a large-scale canine microbiome sequencing survey comparing healthy dogs to those with gastrointestinal disease (18). Host-adapted microbial strains have been found to colonize and interact with the existing host microbiome more effectively than non-native strains (16), and the species included in this study have previously been associated with healthy states (1, 32, 35). *P. hiranonis* plays a role in secondary bile acid conversion, which supports many physiological functions and promotes colonization resistance against pathogens (38, 39). Native *E. faecium* strains have demonstrated stronger mucosal binding than non-native strains, potentially explaining inconsistencies in *in vivo* responses to non-native-based probiotics (40). *M. funiformis* has been linked to healthy states in dogs, humans, chickens, and mice. It produces propionate and acetate and ferments plant matter, including inulin and phytic acid, and a low abundance of *M. funiformis* has been linked to impaired hepatic fatty acid oxidation and certain cardiac conditions in dogs (19, 41). The tolerability and changes observed in clinical outcomes for this cohort of dogs after administration of this novel AMP provide a foundation for future studies assessing its efficacy as a treatment for diarrhea in a controlled setting.

Efficacy of the AMP was not an endpoint of this study, and clinical improvement was monitored to determine which dogs should continue on the AMP for the duration of the study and which dogs should be released from the study to pursue other treatment options; therefore, the cause of diarrhea was not controlled for in this study. GI panel results suggest that the underlying cause of diarrhea in this cohort of dogs was variable and may include pancreatitis. No dogs had serum cTLI values within the diagnostic or equivocal ranges for exocrine pancreatic insufficiency at baseline or throughout the study. The existence of EPI would require additional targeted treatment with pancreatic enzyme replacement therapy to improve associated diarrhea. Four total dogs (Dogs 2, 5, 6, and 9) were found to have cTLI values above the reference range during the study, including two with cTLI values above the reference range at baseline, and two more who showed elevated levels during the treatment period. Serum cTLI concentrations >50 μg/L can be seen in some healthy dogs but can also be increased in dogs with pancreatitis or renal insufficiency. Dogs 5 and 6 were found to have elevated cTLI concentrations at one time point, but all other tested serum components were within ranges considered healthy, and these dogs maintained PFS ≤ 3 during the treatment period. We cannot rule out renal insufficiency or pancreatitis as a cause for elevated cTLI in these dogs.

Similar to EPI, pancreatitis can lead to diarrhea. Four total dogs (Dogs 1, 2, 4, and 9) had serum cPLI values above the laboratory reference interval, including two that had cTLI tests above the reference range. Three of these dogs had a test with elevated cPLI concentrations during the treatment period; however, these changes are unlikely to be attributable to the AMP, as this elevation was transient for two dogs who returned to normal values by day 56, and the third dog exhibited no other symptoms consistent with pancreatitis, including diarrhea (PFS ≤ 3). Dog 4 had normal serum cTLI but elevated cPLI at screening, but was classified as a non-responder and removed before day 7. Dog 9 had a normal cTLI concentration on day 0 but showed elevated serum cTLI and cPLI values above the reference range on day 7, along with an increase in PFS to 4. This may indicate that the dog developed pancreatitis during the study; however, the elevated concentration was in the equivocal range for pancreatitis, and the PFS and cPLI concentrations for Dog 9 returned to normal for the remainder of the treatment period. Pancreatitis is often self-limiting, and clinical signs resolve with time and supportive care, depending on the severity. Dog 2 had cTLI persistently >50 μg/L throughout the study and an elevated cPLI concentration consistent with pancreatitis on day 7 of the treatment period. This dog returned to having a PLI value within the healthy reference interval at day 56. Dog 1 also showed high cTLI values on days 7 and 56 and an elevated serum cPLI concentration consistent with pancreatitis on day 56. However, both Dog 1 and Dog 2 maintained fecal scores between 2 and 3 throughout the treatment period. Because this study was designed to assess tolerability and lacked a control group, definitive conclusions cannot be drawn regarding the effect of the AMP on GI panel values or the observed changes over the treatment period.

GI panel results suggest that the underlying cause of diarrhea in this cohort of dogs may also include distal small intestinal malabsorption. Five dogs in the study (Dogs 3, 4, 9, 10, and 11) had cobalamin or folate concentrations suggestive of distal small intestinal malabsorption at screening. All five of these dogs had serum cobalamin concentrations that were low or near the lower end of the reference range (<400 ng/L), for which exogenous cobalamin supplementation is recommended (37, 42). Three out of the four non-responders were in this group, and two of these non-responders discontinued the study by day 7. Possible underlying gastrointestinal disease suggests that non-responder dogs may have required additional therapies or time for improvement. Nonetheless, Dogs 3 and 10 maintained a healthy fecal score (≤3) despite consistently low serum folate (Dog 3) or folate values that fluctuated below and then above the reference intervals during the treatment period. The observed variety of possible diseases underlying diarrhea presents a confounding factor in this study, so future studies should aim to identify and take into consideration the underlying causes of diarrhea in the study population to avoid confounding efficacy results across different GI conditions.

A majority of dogs had an improvement in their PFS (PFS < 4) after AMP administration. Most non-responders were among the largest dogs, raising the possibility of dose-dependent responses in the smaller dogs. The AMP dose (CFU/g/day) was derived from estimations of total CFUs in the small intestine of healthy Beagles. While this approach provided a standardized starting point, guidance on probiotic dosing is lacking, and despite the 10^7^-CFU magnitude of the doses provided, it may not have scaled optimally for larger body sizes. While live probiotics are not subject to the same pharmacokinetic considerations as small molecules, body size does not typically translate linearly to dosage (43). For example, evidence suggests that intestinal volume does not scale equivalently with body weight (44). In addition, there is no clear guidance on how to account for a strain’s unique abilities to colonize, replicate, and perform activities in its environmental niche. In this study, dogs that were at least 40 pounds were given 2 doses daily, and dogs less than 40 pounds were given 1 dose daily. It is possible that dogs exceeding 75 pounds may require an even higher dose to reach target abundances. The only small non-responder (16 lbs.; Dog 10) remained enrolled because of clinical improvement after AMP initiation despite not meeting the PFS responder threshold. No clear conclusions can be made regarding whether dose affected outcomes in this study, but testing alternate dosing protocols should be considered in future studies.

Acute diarrhea is commonly self-limiting in dogs, so it is not possible to separate any effect of the AMP from typical patterns of microbiome changes in self-limiting diarrhea. However, because the administered AMP is hypothesized to affect the composition of the gut microbiome, stool samples serving as a non-invasive proxy of the lower GI tract (45) were sequenced for an exploratory analysis of microbiome dynamics during AMP administration. Healthy dogs generally exhibit higher fecal alpha-diversity than dogs with gastrointestinal disorders (32), and within this cohort, six out of the seven participants enrolled for at least 7 days showed increases in alpha-diversity metrics. The overall increase in diversity, as measured by Shannon’s H, can be attributed to increased evenness (Pielou’s J), which might be observed during the resolution of an acute dysbiosis episode characterized by few highly abundant taxa. To place diversity changes in a larger context, a reference cohort of 150 previously published microbiome sequencing samples, including healthy dogs and dogs with IBD, was incorporated in beta-diversity analyses. Using this reference, five out of the seven participants demonstrated compositional shifts toward the healthy reference group over the study period. The variation in beta-diversity trends, despite trends toward a healthy GI state as measured by fecal scores and alpha-diversity, may be due in part to the diverse health backgrounds of the dogs included in the pilot study, as previously discussed. Changes in the gut microbiome are associated with diseases affecting the GI tract with or without concurrent diarrhea (46, 47). While the AMP was tolerated by dogs with different drivers of diarrhea, future clinical studies should test efficacy in dogs with specifically defined disease backgrounds.

While species-level dynamics are challenging to assess at the individual level due to the compositional nature of microbiome sequencing data, proportionality ratios of strains can be used to assess changes and provide meaningful insight (33). We observed that the ratio of health-associated to CE-associated taxa used in the Dysbiosis Index (35) increased over the treatment period in six out of seven participants, reflecting a shift toward a microbial profile more typical of healthy dogs. This increase was observed primarily in study patients who had a higher proportion of the CE-associated taxa at the baseline screening (log-ratio < 0), suggesting that this index is most relevant in subsets of dogs with diarrhea that is driven by specific microbiome disruptions.

Although this pilot study represented a small sample of dogs with a diverse study population, including different ages, breeds, base diets, and underlying causes of diarrhea, all study dogs tolerated AMP treatment well. Among dogs that showed a drop in fecal scores by day 7, most maintained these low scores at all following time points, while Shannon diversity increased by day 7 and was sustained at day 56. However, cases of acute diarrhea are commonly self-limiting, and the lack of a control group in the present study prevents any assessment of the AMP’s role in these outcomes. Furthermore, the definition of “chronic diarrhea” was unstandardized, and the exact duration for each dog could not be confirmed, which may have influenced the likelihood of response. The study endpoints relied on subjective assessment of stool photos and on accurate histories from dog owners. Since the study treatment was administered from home, we relied on owners to adhere to instructions for correct dosage and daily use. Given these constraints, the observed improvements in PFS and associated microbiome shifts support further investigation in a randomized controlled trial that targets populations using narrower inclusion criteria based on the underlying causes of diarrhea. Dose-ranging studies will be important to further explore the relationship between CFU and body size and to determine whether optimizing the dose can enhance response rates. This study highlights the possibility of a novel probiotic in the role of treating canine diarrhea and supports promising alternative therapies to conventionally used antimicrobials that have been shown to have potential long-term, detrimental impacts on the gut microbiome. Ultimately, larger controlled trials across standardized patient populations are needed to determine efficacy and assess whether shifting the microbiome composition translates into consistent clinical benefit.

## Data Availability

Demultiplexed sequencing files generated in this study have been deposited in the NCBI Sequence Read Archive under BioProject accession number PRJNA1357358.

## References

[1] Pilla R Suchodolski JS. The role of the canine gut microbiome and metabolome in health and gastrointestinal disease. Front Vet Sci. (2020) 6:498. doi: 10.3389/fvets.2019.00498, 31993446 PMC6971114

[2] Stavroulaki EM Suchodolski JS Xenoulis PG. Effects of antimicrobials on the gastrointestinal microbiota of dogs and cats. Vet J. (2023) 291:105929. doi: 10.1016/j.tvjl.2022.105929 36427604

[3] Ziese A-L Suchodolski JS. Impact of changes in gastrointestinal microbiota in canine and feline digestive diseases. Vet Clin North Am Small Anim Pract. (2021) 51:155–69. doi: 10.1016/j.cvsm.2020.09.004, 33131916

[4] Pilla R Suchodolski JS. The gut microbiome of dogs and cats, and the influence of diet. Vet Clin North Am Small Anim Pract. (2021) 51:605–21. doi: 10.1016/j.cvsm.2021.01.002, 33653538

[5] Velázquez OC Rombeau JL. Butyrate: potential role in Colon Cancer prevention and treatment In: Kritchevsky D Bonfield C, editors. Dietary Fiber in health and disease. Advances in experimental medicine and biology. Boston, MA: Springer US (1997). 169–81. 9361842

[6] Suchodolski JS. Analysis of the gut microbiome in dogs and cats. Vet Clin Pathol. (2022) 50:6–17. doi: 10.1111/vcp.13031, 34514619 PMC9292158

[7] Zhang Y-J Li S Gan R-Y Zhou T Xu D-P Li H-B. Impacts of gut Bacteria on human health and diseases. Int J Mol Sci. (2015) 16:7493–519. doi: 10.3390/ijms16047493, 25849657 PMC4425030

[8] Stone NE Nunnally AE Jimenez V Cope EK Sahl JW Sheridan K . Domestic canines do not display evidence of gut microbial dysbiosis in the presence of Clostridioides (Clostridium) difficile, despite cellular susceptibility to its toxins. Anaerobe. (2019) 58:53–72. doi: 10.1016/j.anaerobe.2019.03.017, 30946985

[9] Kim B Wang Y-C Hespen CW Espinosa J Salje J Rangan KJ . *Enterococcus faecium* secreted antigen A generates muropeptides to enhance host immunity and limit bacterial pathogenesis. eLife. 8:e45343. doi: 10.7554/eLife.45343, 30969170 PMC6483599

[10] Hernández-González JC Martínez-Tapia A Lazcano-Hernández G García-Pérez BE Castrejón-Jiménez NS. Bacteriocins from lactic acid bacteria. A powerful alternative as antimicrobials, probiotics, and immunomodulators in veterinary medicine. Animals. (2021) 11:979. doi: 10.3390/ani11040979, 33915717 PMC8067144

[11] White R Atherly T Guard B Rossi G Wang C Mosher C . Randomized, controlled trial evaluating the effect of multi-strain probiotic on the mucosal microbiota in canine idiopathic inflammatory bowel disease. Gut Microbes. (2017) 8:451–66. doi: 10.1080/19490976.2017.1334754, 28678609 PMC5628651

[12] Suez J Zmora N Zilberman-Schapira G Mor U Dori-Bachash M Bashiardes S . Post-antibiotic gut mucosal microbiome reconstitution is impaired by probiotics and improved by autologous FMT. Cell. (2018) 174:1406–1423.e16. doi: 10.1016/j.cell.2018.08.047, 30193113

[13] Éliás AJ Barna V Patoni C Demeter D Veres DS Bunduc S . Probiotic supplementation during antibiotic treatment is unjustified in maintaining the gut microbiome diversity: a systematic review and meta-analysis. BMC Med. (2023) 21:262. doi: 10.1186/s12916-023-02961-0, 37468916 PMC10355080

[14] Szajewska H Scott KP de Meij T Forslund-Startceva SK Knight R Koren O . Antibiotic-perturbed microbiota and the role of probiotics. Nat Rev Gastroenterol Hepatol. (2025) 22:155–72. doi: 10.1038/s41575-024-01023-x, 39663462

[15] Schmitz S Suchodolski J. Understanding the canine intestinal microbiota and its modification by pro-, pre- and synbiotics—what is the evidence? Vet Med Sci. (2016) 2:71–94. doi: 10.1002/vms3.17, 29067182 PMC5645859

[16] Russell B. J. Brown S. D. Saran A. R. Mai I. Lingaraju A. Siguenza N. . Intestinal transgene delivery with native *E. coli* chassis allows persistent physiological changes. Elsevier Inc. (2021) 10.1016/j.cell.2022.06.050 PMC9464905 35931082

[17] Chen X-J Wang Z-Q Zhou Z-Y Zeng N-Y Huang Q-F Wang Z-W . Characterization of Peptacetobacter hominis gen. Nov., sp. nov., isolated from human faeces, and proposal for the reclassification of *Clostridium hiranonis* within the genus Peptacetobacter. Int J Syst Evol Microbiol. (2020) 70:2988–97. doi: 10.1099/ijsem.0.003925, 32369000

[18] Zengler K Embree M. Methods, apparatuses, and systems for analyzing microorganism strains from complex heterogeneous communities, predicting and identifying functional relationships and interactions thereof, and selecting and synthesizing microbial ensembles based thereon. (2016) Available online at: https://patents.google.com/patent/US20160376627A1/en [Accessed September 3, 2025]

[19] Yang X Zhang M Liu Y Wei F Li X Feng Y . Inulin-enriched *Megamonas funiformis* ameliorates metabolic dysfunction-associated fatty liver disease by producing propionic acid. Npj Biofilms Microbiomes. (2023) 9:84. doi: 10.1038/s41522-023-00451-y, 37925493 PMC10625582

[20] Probiotic use alongside antibiotics. [Clinical Briefs]. International scientific Association for Probiotics and Prebiotics. (2024). Available online at: [https://isappscience.org/for-consumers/infographics/]

[21] Hooda S Minamoto Y Suchodolski JS Swanson KS. Current state of knowledge: the canine gastrointestinal microbiome. Anim Health Res Rev. (2012) 13:78–88. doi: 10.1017/S1466252312000059, 22647637

[22] Kararli TT. Comparison of the gastrointestinal anatomy, physiology, and biochemistry of humans and commonly used laboratory animals. Biopharm Drug Dispos. (1995) 16:351–80. doi: 10.1002/bdd.2510160502, 8527686

[23] LeBlanc AK Atherton M Bentley RT Boudreau CE Burton JH Curran KM . Veterinary cooperative oncology group—common terminology criteria for adverse events (VCOG-CTCAE v2) following investigational therapy in dogs and cats. Vet Comp Oncol. (2021) 19:311–52. doi: 10.1111/vco.12677, 33427378 PMC8248125

[24] Apprill A McNally S Parsons R Weber L. Minor revision to V4 region SSU rRNA 806R gene primer greatly increases detection of SAR11 bacterioplankton. Aquat Microb Ecol. (2015) 75:129–37. doi: 10.3354/ame01753

[25] Parada AE Needham DM Fuhrman JA. Every base matters: assessing small subunit rRNA primers for marine microbiomes with mock communities, time series and global field samples. Environ Microbiol. (2016) 18:1403–14. doi: 10.1111/1462-2920.13023, 26271760

[26] Martin M. Cutadapt removes adapter sequences from high-throughput sequencing reads. EMB Net J. (2011) 17:10. doi: 10.14806/ej.17.1.200

[27] Bolyen E Rideout JR Dillon MR Bokulich NA Abnet CC Al-Ghalith GA . Reproducible, interactive, scalable and extensible microbiome data science using QIIME 2. Nat Biotechnol. (2019) 37:852–7. doi: 10.1038/s41587-019-0209-9, 31341288 PMC7015180

[28] Martino C Morton JT Marotz CA Thompson LR Tripathi A Knight R . A novel sparse compositional technique reveals microbial perturbations. mSystems. (2019) 4:165–8. doi: 10.1128/msystems.00016-19 PMC6372836 30801021

[29] Oksanen J Simpson GL Blanchet FG Kindt R Legendre P Minchin PR . Vegan: Community ecology package. (2001)

[30] Bates D Mächler M Bolker B Walker S. Fitting linear mixed-effects models using lme4. J Stat Softw. (2015) 67:1–48. doi: 10.18637/jss.v067.i01

[31] Lenth RV. Least-squares means: the R package lsmeans. J Stat Softw. (2016) 69:1–33. doi: 10.18637/jss.v069.i01

[32] Vázquez-Baeza Y Hyde ER Suchodolski JS Knight R. Dog and human inflammatory bowel disease rely on overlapping yet distinct dysbiosis networks. Nat Microbiol. (2016) 1:16177. doi: 10.1038/nmicrobiol.2016.177, 27694806

[33] Gloor GB Macklaim JM Pawlowsky-Glahn V Egozcue JJ. Microbiome datasets are compositional: and this is not optional. Front Microbiol. (2017) 8:2224. doi: 10.3389/fmicb.2017.02224, 29187837 PMC5695134

[34] Rognes T Flouri T Nichols B Quince C Mahé F. VSEARCH: a versatile open source tool for metagenomics. PeerJ. (2016) 4:e2584. doi: 10.7717/peerj.2584, 27781170 PMC5075697

[35] AlShawaqfeh M Wajid B Minamoto Y Markel M Lidbury J Steiner J . A dysbiosis index to assess microbial changes in fecal samples of dogs with chronic inflammatory enteropathy. FEMS Microbiol Ecol. (2017) 93. doi: 10.1093/femsec/fix136, 29040443

[36] Edgar RC. Accuracy of taxonomy prediction for 16S rRNA and fungal ITS sequences. PeerJ. (2018) 6:e4652. doi: 10.7717/peerj.4652, 29682424 PMC5910792

[37] Cobalamin Information. Gastrointest Lab. Available online at: https://vetmed.tamu.edu/gilab/research/cobalamininformation/ [Accessed September 3, 2025]

[38] Vidal JE Wier MN Angulo-Zamudio AU McDevitt E Jop Vidal AG Alibayov B . Prophylactic inhibition of colonization by *Streptococcus pneumoniae* with the secondary bile acid metabolite deoxycholic acid. Infect Immun. (2021) 89:e00463-21. doi: 10.1128/IAI.00463-21, 34543118 PMC8594607

[39] He Z Ma Y Yang S Zhang S Liu S Xiao J . Gut microbiota-derived ursodeoxycholic acid from neonatal dairy calves improves intestinal homeostasis and colitis to attenuate extended-spectrum β-lactamase-producing enteroaggregative *Escherichia coli* infection. Microbiome. (2022) 10:79. doi: 10.1186/s40168-022-01269-0, 35643532 PMC9142728

[40] Hanifeh M Spillmann T Huhtinen M Sclivagnotis YS Grönthal T Hynönen U. Ex-vivo adhesion of Enterococcus faecalis and *Enterococcus faecium* to the intestinal mucosa of healthy beagles. Animals. (2021) 11:3283. doi: 10.3390/ani11113283, 34828014 PMC8614307

[41] Li Q Larouche-Lebel É Loughran KA Huh TP Suchodolski JS Oyama MA. Gut dysbiosis and its associations with gut microbiota-derived metabolites in dogs with myxomatous mitral valve disease. mSystems (2021) 6:10.1128/msystems.00111-21. doi:10.1128/msystems.00111-21, 33879495 PMC8546968

[42] Devalia V Hamilton MS Molloy AM the British Committee for Standards in Haematology. Guidelines for the diagnosis and treatment of cobalamin and folate disorders. Br J Haematol. (2014) 166:496–513. doi: 10.1111/bjh.12959, 24942828

[43] Sharma V McNeill JH. To scale or not to scale: the principles of dose extrapolation. Br J Pharmacol. (2009) 157:907–21. doi: 10.1111/j.1476-5381.2009.00267.x, 19508398 PMC2737649

[44] Weber M Aimargues F Paris F. Influence of size on the dog’s digestive function. Bull Acad Vet Fr. (2006) 159:327. doi: 10.4267/2042/47851

[45] Suzuki TA Nachman MW. Spatial heterogeneity of gut microbial composition along the gastrointestinal tract in natural populations of house mice. PLoS One. (2016) 11:e0163720. doi: 10.1371/journal.pone.0163720, 27669007 PMC5036816

[46] Rutgers HC Batt RM Elwood CM Lamport A. Small intestinal bacterial overgrowth in dogs with chronic intestinal disease. J Am Vet Med Assoc. (1995) 206:187–93. doi: 10.2460/javma.1995.206.02.187, 7751219

[47] Isaiah A Parambeth JC Steiner JM Lidbury JA Suchodolski JS. The fecal microbiome of dogs with exocrine pancreatic insufficiency. Anaerobe. (2017) 45:50–8. doi: 10.1016/j.anaerobe.2017.02.010, 28223257

