# Correction: Pilot study evaluating tolerability and changes in fecal microbiota associated with novel probiotic administration to dogs with diarrhea

**DOI:** 10.3389/fvets.2026.1981863

**Published:** 2026-09-18

**Authors:** Jessi Doshier, Brooke Anderson, Fan Yang, Samuel D. Stewart, Kayla A. Calapa, Rachel Cooper, Heather Wilson-Robles, Mallory Embree, Chand Khanna

**Affiliations:** 1 Veterinary Specialty Hospital of San Diego, Internal Medicine, NVA/Ethos, San Diego, CA, United States; 2 Native Microbials, San Diego, CA, United States; 3 Ethos Discovery, Woburn, MA, United States; 4 Massachusetts Veterinary Referral Hospital, Internal Medicine, Woburn, MA, United States

**Keywords:** chronic, diarrhea, dog, microbiome, microbiota, probiotic, tolerability

There was a mistake in Figure 2 as published.

Panel C contained an incorrect taxonomic annotation. The strain ASCUSCA103 was erroneously labeled as *Enterococcus faecalis*; the correct taxonomic assignment is *Enterococcus faecium*, consistent with its identification elsewhere in the article.

The corrected Figure 2 appears below.

**Figure 2:**
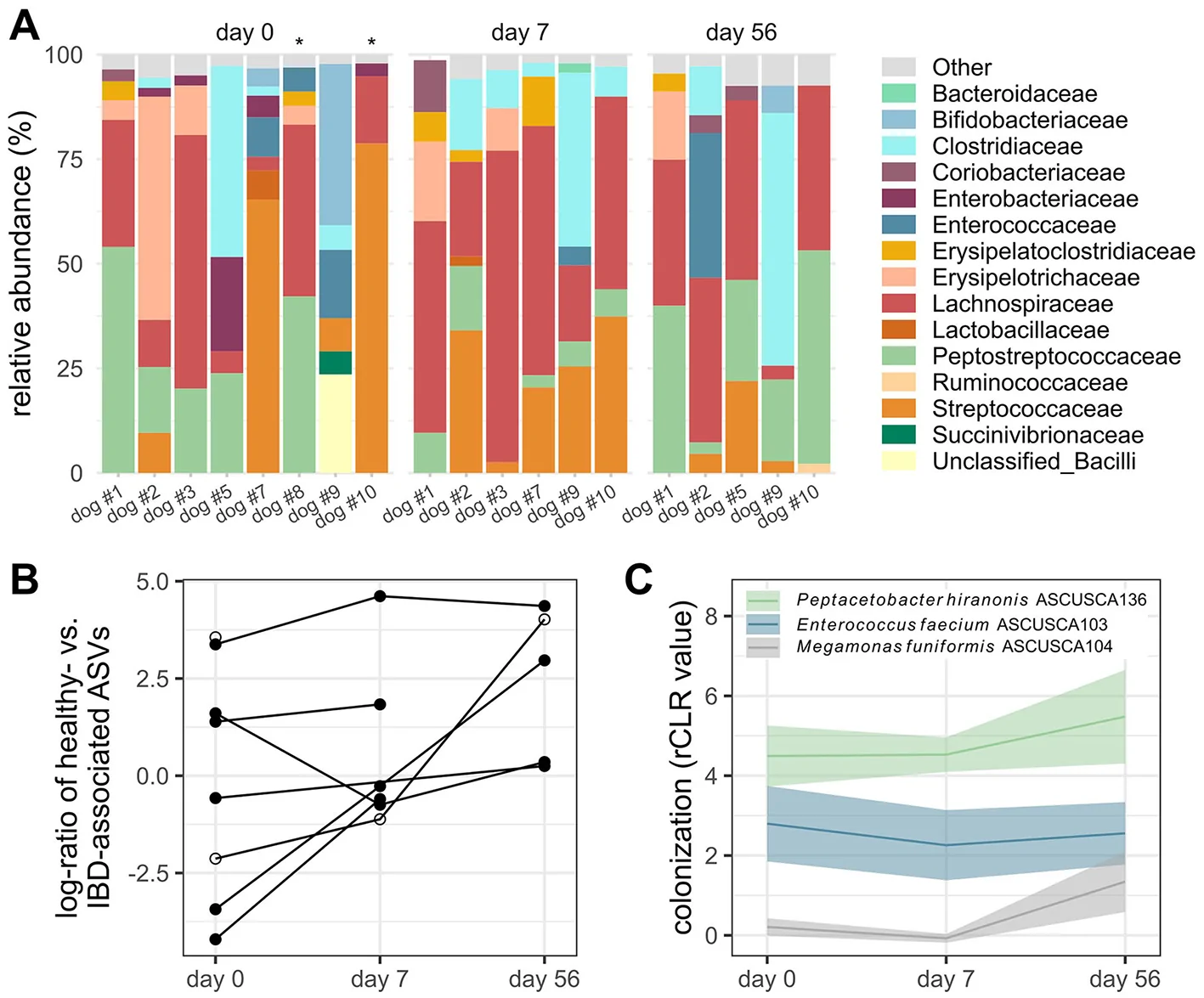
Taxonomic characterization of each dog's fecal samples at each collected time point. **(A)** Family-level relative abundance of ASVs in fecal samples collected during the screening visit (day 0) and after 7 and 56 days of AMP treatment. Asterisks mark screening samples from dogs later classified as non-responders. **(B)** Changes in the ratio of ASVs that align to taxa used in the dysbiosis index (18), expressed as a log-ratio of ASVs more common in healthy dogs to those more common in CE/IBD dogs (see Methods section). The ratio is computed as summed reads from *Fusobacterium, Faecalibacterium, Turicibacter, Blautia*, and *Peptacetobacter hiranonis* divided by summed reads from *Streptococcus* and *Escherichia coli*. Open symbols indicate non-responders. **(C)** Abundance of the administered AMP strains, quantified as the mean rCLR-transformed value of ASVs with 100% identity to the sequenced 16S rRNA gene region of each canine-derived strain included in the AMP.

The original version of this article has been updated.

